# Perspectives Among Veterans with Chronic Pain and Co-Occurring Mild Traumatic Brain Injury: Mixed-Method Findings from a Neuromodulation and Yoga Intervention

**DOI:** 10.3390/ijerph23070872

**Published:** 2026-07-03

**Authors:** Amy M. Kemp, Kelly Krese, Bella Etingen, Bridget A. Cotner, Sadie Walker, Ibuola Kale, Miriam R. Rafferty, Sandra Kletzel, Rachana P. Shah, Sabrina Bedo, Sarmistha Chaudhuri, Alexandra L. Aaronson, Kyla Z. Donnelly, Sonia Bobra, Andrea Billups, Pei-Shan Yen, Dulal Bhaumik, Theresa L. Bender Pape, Amy A. Herrold

**Affiliations:** 1 Elson S. Floyd College of Medicine, Washington State University, Spokane, WA 99202, USA; 2 Edward Hines Jr. VA Hospital, Hines, IL 60141, USA; bridget.cotner@va.gov (B.A.C.); sadie.walker@va.gov (S.W.); ibuola.kale@va.gov (I.K.); sandra.kletzel@va.gov (S.K.); rachana.shah@va.gov (R.P.S.); sabrina.bedo@va.gov (S.B.); sarmistha.chaudhuri@va.gov (S.C.); alexandra.aaronson@va.gov (A.L.A.); kyla@loveyourbrain.com (K.Z.D.); sbobra@uic.edu (S.B.); andrea.billups-caldwell@va.gov (A.B.); amy.herrold@va.gov (A.A.H.); 3 Shirley Ryan AbilityLab, Chicago, IL 60611, USA; kkrese@midwestern.edu (K.K.); mrafferty@sralab.org (M.R.R.); t-pape@northwestern.edu (T.L.B.P.); 4 Midwestern University, Downers Grove, IL 60515, USA; 5 Research and Development Service, Dallas VA Medical Center, Dallas, TX 75216-7167, USApeishan0824@gmail.com (P.-S.Y.); dbhaumik@uic.edu (D.B.); 6 Department of Health Economics, Systems, and Policy, Peter O’Donnell Jr. School of Public Health, UT Southwestern Medical Center, Dallas, TX 75390, USA; 7 Morsani College of Medicine, University of South Florida, Tampa, FL 33612, USA; 8 Feinberg School of Medicine, Northwestern University, Chicago, IL 60611, USA; 9 Department of Psychiatry, College of Medicine, University of Illinois Chicago, Chicago, IL 60612, USA

**Keywords:** mild traumatic brain injury, chronic pain, intermittent theta burst stimulation, lived experience

## Abstract

**Highlights:**

**Public health relevance—How does this work relate to a public health issue?**

- Chronic pain and mild traumatic brain injury (mTBI) are highly prevalent, co-occurring conditions among Veterans that contribute substantially to long-term disability, reduced quality of life, and increased healthcare utilization.
- There is a critical need for effective, scalable, non-pharmacological interventions to address multidimensional symptoms (physical, emotional, and social) while reducing reliance on medication-based pain management strategies.

**Public health significance—Why is this work of significance to public health?**

- This study provides support for a combined neuromodulation (iTBS) and mind–body (yoga) intervention as an approach to improving pain-related outcomes, emotional health, and self-management in Veterans with chronic pain.
- The findings highlight that meaningful improvements in patient-centered outcomes—such as self-efficacy, coping, and social engagement—may not be fully captured by standard clinical metrics, underscoring the need for more comprehensive outcome assessment in public health research.

**Public health implications—What are the key implications or messages for practitioners, policy makers, and/or researchers?**

- Integrated, non-pharmacologic interventions that combine neuromodulation with behavioral or mind–body therapies may offer a scalable, patient-centered model for managing chronic pain and co-occurring conditions within healthcare systems, such as the VA.
- Future public health efforts should prioritize (1) broader implementation and rigorous evaluation of multimodal interventions and (2) the inclusion of patient-reported and qualitative outcomes to better capture real-world impact and inform policy, clinical guidelines, and resource allocation.

**Abstract:**

Chronic pain is the leading cause of disability worldwide and frequently co-occurs with mild traumatic brain injury among Veterans (mTBI + CP), creating complex treatment challenges and a need for novel, non-pharmacological interventions. This study evaluated a pilot intervention combining intermittent theta burst stimulation (iTBS), a neuromodulatory approach, with the evidence-based LoveYourBrain Yoga program to enhance rehabilitation outcomes. In a six-week open-label trial, ten Veterans with mTBI + CP received weekly iTBS followed by yoga sessions. Pilot quantitative outcomes included quality of life (Traumatic Brain Injury Quality of Life [TBI-QoL]) and functional ability (Mayo Portland Adaptability Inventory-4 [MPAI-4]), assessed pre- and post-intervention, alongside qualitative semi-structured interviews and interdisciplinary clinical notes. Significant improvements were observed in TBI-QoL Fatigue (*p* = 0.021) and MPAI-4 Grief and Loss (*p* = 0.016), with clinically meaningful but non-significant gains in Ability and Adjustment. Qualitative findings revealed improved pain management and enhanced self-management, with participants describing better emotional regulation, more effective coping strategies, and stronger social connections. Some benefits were more evident in qualitative data than in standardized measures. These pilot findings suggest that combining iTBS with mind–body therapy may provide complementary tools for pain management and functional recovery in Veterans with mTBI + CP, supporting further investigation of integrated neuromodulation and behavioral interventions.

## 1. Introduction

Mild traumatic brain injury (mTBI) and chronic pain frequently co-occur. For example, of the 340,000 individuals who sustained mTBI related to military conflicts in Iraq and Afghanistan [1], up to 70% are also estimated to have chronic pain [2,3]. Chronic pain impairs physical and psychological function [4,5,6] and decreases quality of life, and these impacts are exacerbated among those with co-occurring mTBI [7]. Chronic pain and mTBI are both conditions categorized as “invisible wounds” that profoundly shape daily functioning yet remain unseen by others [8,9]. These challenges are further compounded by stigma surrounding help seeking and ingrained expectations of self-reliance within military culture [10,11,12,13,14]. As a result, active-duty service members and military Veterans may struggle privately with physical or emotional pain, cognitive disruptions, and difficulty identifying and fulfilling social roles [15,16].

In recent years, recommendations around pain management have shifted away from medication-based treatment [17,18,19]. Accordingly, effective, non-pharmacological treatment options are needed to address chronic pain and related sequelae [20]. Yoga is a complementary and integrative health therapy that incorporates physical movement, breathing exercises, and meditation practices. Clinical practice guidelines and systematic reviews recommend yoga as a non-pharmacological treatment for reducing chronic pain and improving quality of life [18,21]. Research also indicates that yoga is safe and effective for individuals with neurological conditions [22].

LoveYourBrain Yoga (LYB Yoga) is a standardized and manualized, group-based yoga program that incorporates gentle physical postures (asanas), breathing exercises (pranayama), meditation, and psychoeducation components [23] which are specifically designed for use among individuals with mTBI [24]. Studies focused on LYB Yoga indicate that participation in this intervention is associated with improved quality of life among individuals with TBI [24]. The psychoeducation component of LYB Yoga focuses on the transdiagnostic theme of resilience and is intended to help participants learn to manage, cope with, and better understand their health conditions [23]. Yoga is also thought to offer neuroplasticity benefits [22,25], which posits it as an optimal intervention to pair with neuromodulation to improve function and quality of life [26,27].

Transcranial magnetic stimulation (TMS) is a non-invasive brain stimulation technique used to modulate neural activity. The use of a high-frequency, excitatory repetitive transcranial magnetic stimulation (rTMS) delivered to the motor cortex is an effective method for reducing pain [28,29]. rTMS provided to the primary motor cortex (M1) induces neuromodulation of the descending pain pathways involved with inhibiting nociception [30,31]. Intermittent theta burst stimulation (iTBS) is a 3 min TMS protocol that utilizes a patterned excitatory rTMS, creating a window of enhanced excitability for at least 60 min, thereby offering an opportunity to enhance the benefits of targeted activity-based interventions provided afterwards [32]. For example, iTBS provided prior to high frequency (10 Hz) rTMS to the M1 provided greater analgesia than rTMS alone (without iTBS priming) among patients with chronic refractory neuropathic pain [33,34]. iTBS was successfully combined with cognitive–behavioral therapy to promote smoking cessation [34].

As outlined above, rTMS and yoga each alone improve pain outcomes [21,28,29], as well as modulate pain pathways [31,35], and iTBS, specifically, induces a window of neuroplasticity [32]. Thus, we developed an intervention that combined iTBS immediately followed by small-group LYB Yoga [36]. Additional information on the rationale for the combined treatments can be found in the protocol paper [36] and in a more in-depth article published on these treatments for chronic pain [37]. Our team found that the iTBS + yoga intervention was safe, feasible, and acceptable, and participation was associated with improved pain intensity among Veterans with mTBI and chronic pain [38].

Given the novelty of this combined intervention, the objective of the current analysis was to examine qualitative and quantitative perspectives of Veterans on the pilot iTBS + yoga intervention. Specifically, we explored patient-reported outcomes and qualitative experiences among Veterans who completed the intervention to evaluate function and quality of life outcomes as perceived by the Veterans. Findings from this small pilot sample are descriptive and intended to support the Veteran experience and inform future protocols for iTBS + yoga interventions.

## 2. Materials and Methods

This study is reported in accordance with the reporting guidelines for mixed-methods reporting in rehabilitation and health science (MMR-RHS) [39] and the CONSORT extension for feasibility trials checklist. The purpose of this study was to describe qualitative and quantitative perspectives of Veterans on the integrated iTBS + yoga intervention. Specifically, qualitative interviews were used to characterize Veterans’ experiences, perceived benefits, acceptability, and feasibility of intervention, while quantitative patient-reported outcomes were examined descriptively to provide additional context for Veteran-reported experiences. Findings from the study should, therefore, be interpreted as preliminary, descriptive, and hypothesis-generating, and not as evidence of intervention effectiveness.

Ethical approval for this project was obtained from the VA IRB (IRB# 20-013). This study also operated under an FDA Investigational Device Exemption (G200195) and is registered in clinicaltrials.gov #NCT04517604. The full study included a pre- and post-measurement session, a 6-week intervention with weekly sessions, and a qualitative interview at the end of the 6 weeks. Participants received $280 as compensation for completing the full intervention.

### 2.1. Participants and Intervention

This paper examines quantitative and qualitative data collected from an open-label, single-group pilot study. All participants received active/real iTBS followed by active/real LYB Yoga. The study protocol, published by Krese et al. [36], details the eligibility criteria (see Supplemental Table S1 in [36]), recruitment procedures, and the iTBS + yoga intervention. Briefly, Veterans met American Congress of Rehabilitation Medicine/VA/Department of Defense criteria for mTBI history [40,41] incurred at least 1 year prior to enrollment as confirmed via a structured clinical interview and reported current symptoms on the neurobehavioral symptom inventory as described in Pape et al. [42], except that cognitive symptoms were not required. Veterans were also eligible if they reported chronic musculoskeletal pain of moderate to severe intensity for more than 6 months, as measured by the Brief Pain Inventory. Individuals with contraindications to MRI or iTBS were excluded.

The intervention consisted of combined iTBS and yoga sessions once a week for six weeks; see Krese et al. [36] for the full protocol. Neuronavigated (Localite TMS Neural Navigator System, Belgium) iTBS was provided over the trunk region of the dominant primary motor cortex (M1). A 3 min iTBS protocol was delivered using a MagPro X100 with MagOption stimulator with a figure-of-eight Cool-B60 or equivalent Cool-B65 A/P coil using the Active setting only (MagVenture, Farum, Denmark). Three pulses of stimulation were given at 50 Hz, repeated every 200 ms at 80% of the resting motor threshold, with an inter-pulse interval of 20 ms. A 2 s train of iTBS was repeated every 10 s for a total of 190 s, for a total of 600 pulses [36]. Data safety monitoring sheets that collected 17 safety indicators, including vital signs, headache, and tinnitus, were collected before, during, and after each iTBS session and reported in previous reports (Supplemental Digital Content 1) [38]. Adverse events were also collected on an adverse events log and reported previously [38]. Immediately after iTBS, participants transitioned to small-group LYB Yoga sessions, which consisted of 10 min of breathwork, 45 min of physical postures, 15 min of meditation, and 20 min of guided psychoeducation.

**Figure 1:**
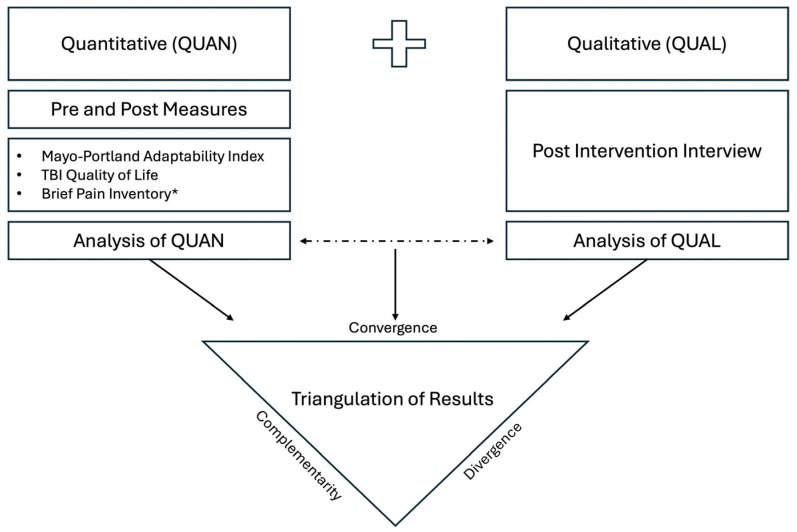
Overview of methods for triangulation in this study. Quantitative data and qualitative analysis concurrently occurred and informed the triangulation of the results. * Brief Pain Inventory outcomes reported in Krese et al. [37].

Nineteen Veterans enrolled in the study; 14 ultimately initiated iTBS + yoga participation; and 10 Veterans completed all intervention sessions (see Krese et al. [36] reference Figure 1 for the study CONSORT diagram). This paper includes quantitative and qualitative data collected from the 10 intervention completers.

### 2.2. Recruitment

Veterans were recruited from a large VA Hospital in the Midwestern US. Study staff distributed IRB-approved study flyers at TBI/polytrauma staff meetings and to Veterans in VA clinics. We also invited previous research participants from our laboratory who are included in an IRB-approved TBI data repository (IRB#14-003, PI Herrold). As part of the study, participants who completed the intervention were invited to participate in a post-intervention qualitative interview to detail their perspectives on the intervention. This interview used a structured guide that reviewed each session of the intervention and asked the participant to reflect on that day’s activities, the outcomes experienced throughout the intervention, and any other observations.

### 2.3. Quantitative Methods

Participants completed self-report measures of function and quality of life before and after receiving the iTBS + yoga intervention. These measures included the Mayo-Portland Adaptability Index (MPAI-4) [43] and quality of life (TBI Quality of Life (TBI-QoL) [44]. Both measures generate subscale scores. The Brief Pain Inventory was also collected and is reported elsewhere [38]. In the current analysis, quantitative patient-reported outcomes were used to provide descriptive context for Veterans’ interviews.

### 2.4. Qualitative Methods

Interviews were conducted with all 10 participants who completed the intervention. Interviews assessed intervention feasibility and acceptability and included broader reflection prompts that examined past reflections for each of the six iTBS and yoga sessions. Author B.E., a trained qualitative researcher, conducted the interviews virtually, with an in-person research team member present at each interview to manage the technology. Interviews were audio-recorded and transcribed verbatim. The interview guide is available in the Supplementary Materials.

### 2.5. Data Analysis

As noted above, this study aimed to explore outcomes and experiences among Veterans who completed the iTBS + yoga intervention. Descriptive statistics were used to characterize the sample’s demographics.

Linear mixed-effects models were employed to evaluate the association between changes in TBI-QOL and MPAI-4 outcomes and time, representing pre- and post-intervention time points. Time was considered a fixed effect in the model to assess the impact of the iTBS + yoga intervention on outcomes. To address within-subject variability from repeated measures over time, a random effect was included in the model. Statistical analysis was conducted in R (version 4.3.0). Statistical significance is determined to be *p* < 0.005.

Atlas.ti (version 25) software was used to organize qualitative data, which were thematically analyzed from a phenomenological perspective using a deductive approach following the phases of analysis outlined by Braun and Clarke [45]. These phases include data familiarization, initial coding, searching, reviewing and defining themes, and reporting the data. All transcripts were reviewed by at least two researchers to ensure accuracy and completeness and were accompanied by field notes as part of a reflective approach [35]. All transcripts were dual-coded and reread to ensure that all relevant data were extracted. Once the themes were formed, all authors met to discuss, compare, and synthesize the data. The themes were evaluated for repetition, and final themes and sub-themes were identified.

Triangulation was used to compare Veteran-reported outcomes with TBI-QoL, MPAI-4 scores, and qualitative themes and to categorize them as convergent, complementary, or divergent. Convergence of similar findings across methods enhances credibility [46,47]. Complementarity highlights the unique strengths of qualitative and quantitative methods, which together contribute to shared outcomes [47]. Divergence indicates differing results, offering opportunities for deeper investigation or new research [46,47]. Please see Figure 1 for an overview of the methods and analysis.

### 2.6. Rigor and Trustworthiness

Authors B.E. and A.K. took field notes during the interviews or after each transcript review to enhance self-reflexivity. Deductive and inductive coding techniques were used to consider all relevant information and minimize bias. Four researchers from diverse professional and research backgrounds were also involved in developing the analytic strategy, executing the analysis, and reporting the results to reduce potential bias. Finally, 30% (*n* = 3) of the transcripts were dual-coded by two trained qualitative researchers, with 98% interrater reliability. Disagreements in coding were resolved through consensus with a third coder.

## 3. Results

We included 10 Veterans who participated in the intervention between August 2021 and August 2023 in our analysis. The majority of participants (70%, 7/10) were male, White (60%, 6/10), and of non-Hispanic ethnicity (80%, 8/10). Average participant age was 46.2 years (std. dev. 11.6). All participants had chronic musculoskeletal pain in one or more locations, including low back pain. Table 1 summarizes participant demographics and pain characteristics. To provide contextual information on quantitative outcomes, both self-reported experiences of participants and function and quality-of-life outcomes are described here.

### 3.1. Quantitative Outcomes

#### 3.1.1. Function

The MPAI-4 produced an overall functioning score and subscale scores in ability, adjustment, and participation. Changes in average MPAI-4 scores (Table 2) among participants were not statistically significantly different from pre- (ability 39.8 avg. ± 11.5 SD.; adjustment 47.0 ± 8.30; participation 29.8 ± 12.2; overall 39.8 ± 10.5) to post-intervention (ability 34.0 ± 10.3; adjustment 41.2 ± 11.5; participation 31.0 ± 5.65; overall 35.2 ± 17.4). Although not statistically significant, we did detect clinically meaningful differences on the MPAI-4 ability and adjustment subscales based on MPAI-4 indices of minimally clinically important differences (MCID; [48]) of 5 t-score points (≈0.5 SD).

#### 3.1.2. Quality of Life

The TBI-QoL comprises 17 subscales (listed in Table 2) with scores ranging from 0 to 100. For certain subscales (e.g., Independence), higher scores indicate better quality of life, while for other scales (e.g., Fatigue, Grief, and Loss), higher scores indicate worse quality of life. Of the 17 areas assessed with the TBI-QoL, average participant scores on the Fatigue (56.8 ± 9.51 vs. 52.5 ± 9.34, mixed effects, *p* = 0.021) and Grief and Loss (45.0 ± 8.23 vs. 41.4 ± 9.56, mixed effects, *p* = 0.016) subscales demonstrated statistically significant changes from pre- to post-participation.

### 3.2. Qualitative Outcomes

Interviews with participants lasted an average of 32 min (range: 22–50 min). Themes were organized into two main categories: (1) pain management, which included two sub-themes (physical health; building an internal toolset), and (2) self-management, which also had two sub-themes (emotional health; social health). These themes and sub-themes provided insights into how participants managed chronic pain and their perceived benefits of the intervention. Please see Table 3 for a list of themes, sub-themes, and codes.

#### 3.2.1. Pain Management

Participants described yoga as providing practical, adaptable strategies for managing pain, particularly through breathing and mindfulness techniques. These are described through two sub-themes: physical health and building an internal toolkit (cognitive health).

##### Physical Health

Interviewees noted that practicing yoga yielded tangible physical health benefits. Participants reported improved movement and mobility: “My experience [showed me] that I was making progress… I couldn’t get full rotation in my shoulder, but now… I got full movement on my shoulder.” (C) Interviewees also described experiencing increased flexibility and reduced stiffness, which reduced interference with activities: “I’ve been noticing that like flexibility wise, I think I got a lot better. My backs not as stiff and doesn’t hurt as bad.” (E) Several participants also noted overall reductions in pain: “I would say the level or the amount of lower back pain has reduced. At some points in the last week, last couple days, I haven’t had any back pain at all.” (I.)

For many, these physical improvements extended into daily life. Yoga was described as enhancing general well-being (“Physically, I felt better. I slept better. I communicate better. All around I think I’m a better person than I was when I started.”—B) and supporting subtle but meaningful changes in physical functioning (“It might be a gradual onset. Hopefully, I’m not fooling myself, but I think I have noticed some small changes in the way I walk….”—J). Some interviewees also noted that reduced pain led to them having less fatigue and more available energy: “I noticed more energy… throughout the day when I did the yoga. One thing about physical pain is that it tires you out, so being able to be less tired throughout the day. I think it was the yoga that helped because I can’t function having that constant pain, so it just gives you that feeling that you’re not wasting all that time on that pain and energy.” (I.)

Finally, participants highlighted how pain relief allowed them to re-engage in and enjoy day-to-day and leisure-time activities. As one Veteran shared, “I was able to just come home and enjoy things.” (I) As another Veteran described: “I made it through an 8-h drive on Saturday and went to an hour concert and was not in agonizing pain like I normally would have been.” (E.)

##### Building an Internal Toolset

Interviewees noted that they valued the skills they learned through LYB Yoga not only for alleviating discomfort but also for promoting a sense of control. One Veteran explained, “Breathing helps calm the system down, more oxygen in your lungs … it helps alleviate the pain and the stress you’re going through.” (C) The portability of these strategies was especially meaningful: “I don’t need any special tools; I can do it wherever I am … something I’ve learned that I can take with me anywhere and use it.” (J) For Veterans who had struggled to find consistent pain management approaches, this flexibility represented a critical shift. Several participants emphasized the importance of taking ownership in adapting activities to fit their abilities: “I just have to adapt what I was doing … Maybe I do tubing instead of water skiing or cut down the amount of time. Find my limitations.” (E) Others highlighted the complementary nature of yoga alongside external interventions: “This type of stimulation takes pain management out of your hands, but the yoga puts it back in my control.” (F) Ultimately, many participants noted that they had enrolled in the study to explore new pain management options and, through their participation, had discovered a renewed sense of control: “That medication ain’t always the solution. … I can escape or relief my pain without a pill or without any kind of medication. And that’s what I was looking for.” (B.)

#### 3.2.2. Self-Management

Interviewees relayed that participation in the intervention taught them practical pain management strategies, such as mindfulness and breathing exercises, along with opportunities for success and validation from peers to help manage life with chronic pain. These tools and experiences helped them develop tangible changes in their routines and relationships, as one Veteran explained, “Being more relaxed at home … not feeling like I have so many things I have to get done, but instead being able to slow down.” (I) Others emphasized how these practices shifted their internal awareness: “… an outlook about their own life … can definitely shift the automatic thoughts or things that pop up that might actually be a detriment to your mental well-being.” (H) For some, this translated into improved emotional control and overall well-being: “I carry a lot of stress, and it just made me hyper-aware that I needed to calm down … Being mindful of what I’m doing and seeing myself clearly has been transformative.” (J) These were overall described in two sub-themes: physical health and emotional health.

##### Physical Health

Participants described that breathing technique, peer support, and portability tools (described above) directly supported their emotional health, including by bolstering their confidence and motivation. For some, physical accomplishments attained during yoga sessions led to increased confidence: “It’s nice to feel a little bit of self-confidence. And I think maybe doing the yoga poses and knowing that I could do them all helped. You know, knowing that my body can do all that.” (G) For some, this confidence also extended into greater motivation to fully participate in the intervention: “I was getting more comfortable with doing the yoga, but then also looking forward to the meditation because the talking, atmosphere, different readings, and the quotes while meditating just really allowed me to focus on the here and now.” (I.)

For some, participating in the intervention prompted feelings of motivation and self-efficacy: “… being able to prove to myself that I had more resilience than I thought I did and, also being able to empathize with others who have similar chronic pain.” (H) For some interviewees, feelings of motivation and self-efficacy led to increased engagement: “I noticed that I did become more active at home. Normally, I kind of push stuff off. But like the last 6 weeks I’ve been really like doing more stuff over the weekends.” (G.)

##### Emotional Health

In addition to facilitating chronic pain self-management, participants described how the intervention fostered meaningful social support, validation, and improved engagement in family and community roles. These connections fostered a sense of community and gratitude, as one Veteran described: “One of the biggest things is the talking that we did throughout sessions with the other guys in the group. That was helpful. And then all the conversations that we had, I left feeling grateful.” (C) Another Veteran noted: “Coming back to the VA, meeting new people, learning more about myself and how it’s okay to share. I don’t have to keep everything bottled up inside … there’s a lot more support for me than I thought.” (I) Participating in the intervention also helped some Veterans validate their experiences and develop coping mechanisms through shared understanding: “Just having [my pain] validated in a room full of people … makes me feel like I’m not alone. … even though I try to be positive sometimes, things get to you. Sometimes you can be your own worst enemy … So it gave me some tools to [be positive].” (J.)

Building on improved emotional health and the sense of connectedness fostered by the program, participants described an enhanced ability to engage in their social roles, including relationships with family and friends. One Veteran reflected, “I expected physical or stress-related [improvements], but I didn’t actually make that correlation, it would make me feel nurturing or able to strengthen my own connections with my social circle, whether it was a friend, my family, or my dogs.” (H) Similarly, others emphasized how the program positively shaped interactions at home: “I noticed within my own household I was a little bit more focused. I get home and my daughter or my wife will ask me how this session was and how is the yoga. I talk about it a little bit more, and it was nice to see I had that support not only here at the VA, but also at home.” (I) Together, these accounts demonstrate how the benefits of yoga and mindfulness extended beyond symptom reduction to support meaningful participation in family, work, and leisure roles.

### 3.3. Triangulation

Integration occurred during the interpretation stage using a side-by-side comparison method (Table 4). Quantitative results were initially analyzed to identify patterns in key outcomes, followed by an examination of qualitative themes to provide context and explain these findings. The results were then compared to identify convergence (where data sources agree), complementarity (where qualitative data elaborates or clarifies quantitative results), and divergence (where findings differ). Convergence was observed in emotional health outcomes, with participants reporting improved resilience, stress regulation, and outlook, which aligned with significant reductions on the TBI-QoL Fatigue (*p* = 0.021) and Grief/Loss (*p* = 0.016) scales. Complementarity was evident in functional areas: the MPAI-4 showed clinically meaningful improvements in the Ability and Adjustment subscales. Qualitative data offered context by describing how participants used mindfulness, breathing, and coping strategies to regain control and confidence in their daily lives. Divergence appeared in emotional and social health outcomes. Although qualitative themes highlighted deep peer validation, family involvement, and stronger social bonds, quantitative measures of social participation did not reflect these improvements. Similarly, participants described increased resilience and empathy, although the TBI-QoL Resilience subscale showed a positive but non-significant trend.

## 4. Discussion

This mixed-methods analysis provides an understanding of how Veterans experienced a novel iTBS + yoga intervention. Through triangulation of similarities and differences across data sources, our findings indicate that Veterans with chronic pain and mTBI experienced benefits extending beyond previously reported reductions in pain severity (Krese et al. [38]). Specifically, participants demonstrated improved pain self-management, enhanced functioning, better quality of life, and greater perceived capability in daily activities. Collectively, these results inform how protocols for iTBS + yoga may address the multidimensional burden of chronic pain by producing integrated gains across physical, emotional, and social domains.

### 4.1. Triangulation Convergence-Improved Quality of Life and Pain Management

Our findings related to pain suggest that iTBS + yoga improved pain outcomes, both in terms of the previously reported reductions in pain severity in Krese et al. [38] and in pain self-management. In line with previous clinical trials [49] and review articles [50], the present study reported meaningful outcomes for participants and supplemented these findings with qualitative interviews, in which participants reported improved ability to manage their chronic pain. Veterans experienced a reduction in pain, improvements in psychological health, and decreased reliance on pharmaceutical treatments in a variety of chronic pain conditions. These are important findings given the priority in Veteran populations to reduce pharmaceutical options [51,52].

An especially notable finding was the reported reduction in grief and loss, as well as in fatigue, outcomes that participants qualitatively described as associated with improved functioning and quality of life. Chronic pain often exacerbates feelings of loss, whether of physical capability, social roles, or personal identity [53,54], and can drain energy reserves, compounding the challenges of daily life with pain [55,56,57]. Participating in iTBS + yoga helped to alleviate these burdens for many participants, enabling them to re-engage with activities, relationships, and self-care practices that had previously felt unattainable.

### 4.2. Triangulation Complementary-Improved Skills and Self-Efficacy

Our observed decrease in grief/loss and fatigue also indicates a broader shift in iTBS + yoga participant perceptions of their own abilities, from focusing on limitations to recognizing opportunities for agency and the capacity to live with chronic pain. This finding is particularly significant in demonstrating that a combination of physiological and behavioral approaches, such as iTBS + yoga, can address not only the symptoms of chronic pain but also the emotional and cognitive factors that can lead to greater self-management and self-efficacy. Previous studies have shown that increasing self-efficacy supports chronic pain self-management [47,48,49]. Yoga alone has been shown to support neurobiological regulation of stress, and since iTBS modulates cortical excitability, it is hypothesized that these effects can be enhanced in combination [28,58].

### 4.3. Triangulation Divergence-Functional and Perceived Abilities

In these findings, the difference between quantitative and qualitative findings was evident in differences in functional and perceived abilities. Quantitative measures, the MPAI-4 and TBI-QoL, captured minimal to no significant improvement in standardized functional outcomes, including mobility, social participation, and role satisfaction. Conversely, qualitative interviews supported substantial improvements in productivity, physical abilities, social engagement, and overall well-being, as reported by participants. In interviews, participants consistently reported meaningful improvements in their ability to manage pain and engage in daily and leisure-time activities, noting direct functional gains such as increased flexibility, reduced pain interference, and enhanced engagement in both social and recreational activities.

One explanation for the observed mismatch may relate to participants’ baseline functional status. Although the MPAI-4 is designed to minimize ceiling effects, individuals with relatively high baseline functioning may show reduced variability on certain Ability items, thereby attenuating sensitivity to change in these domains. In this sample, most participants entered the study with mild-to-moderate functional limitations on the MPAI-4, suggesting limited room for detectable improvement. This interpretation is consistent with prior research [59] indicating that individuals with chronic pain often experience fluctuating and episodic functional limitations rather than stable or severe impairments.

Additionally, these findings may indicate a supplemental finding of increased self-efficacy. Throughout the qualitative interviews, self-efficacy was a key theme for improvement, even though it was not fully reflected in the quantitative data. This is not surprising given that the LYB Yoga program is centered on resilience [24]. Participants reported increased confidence, motivation, and improved emotional well-being, all of which are essential components of self-efficacy [60]. For participants in this study, reported changes in self-efficacy appeared to support chronic pain self-management. The role of self-efficacy in quality of life has been evaluated in other studies [61,62,63], indicating that iTBS + yoga may be a particularly beneficial approach for teaching and improving self-efficacy, thereby enhancing self-management of chronic pain.

### 4.4. Limitations

This study has several limitations. First, the trial was open-label, with all participants receiving the full treatment protocol, including iTBS and yoga. Yoga or iTBS alone was not compared with the combination of iTBS + yoga. Future studies will employ a double-blinded, three-group design to better understand the benefits of the combined iTBS + yoga intervention compared to both iTBS alone and LYB Yoga alone. However, in a protocol development study, it was essential to iteratively develop a feasible and acceptable intervention and to assess its safety, feasibility, and acceptability to prepare it for large-scale, rigorous testing. Second, given the study’s pilot nature, the sample size was limited, and we lacked sufficient power to detect significant changes in secondary outcomes, such as MPAI-4 and TBI-QoL. Although we observed significant differences in some patient-reported outcomes, these findings should be interpreted with caution as pilot and descriptive rather than as evidence of intervention effectiveness. Third, although the sample included some demographic and clinical variability, the small sample size limits conclusions about representativeness and prevents meaningful examination of subgroup differences in Veteran perspectives. Future studies should prioritize targeted recruitment strategies to enhance diversity and inclusion. Finally, interviews were conducted only with those who completed all six iTBS + yoga sessions, highlighting the modest attrition rate (30%) in this study. In turn, this may influence the generalizability of the findings to other populations with varying levels of motivation, pain, and pain management approaches. Future research will make specific efforts to better support Veterans in completing the full study and understand what may impact completion.

### 4.5. Clinical Implications

The clinical implications of this study should be interpreted cautiously as this study analysis was not designed or powered to establish intervention efficacy. Instead, findings provide pilot perspectives of an iTBS + yoga intervention for Veterans with mTBI and chronic musculoskeletal pain. Veterans who completed the intervention described perceived benefits related to pain self-management, fatigue, emotional regulation, and grief/loss, suggesting that these domains may be important outcomes to examine in future trials. Larger controlled studies with more diverse samples and objective functional outcomes are needed before clinical recommendations can be made.

## 5. Conclusions

This study demonstrates that combining iTBS with yoga may provide Veterans with chronic pain a practical, non-pharmacologic approach to both symptom relief and self-management. Participants described acquiring portable, adaptable strategies that reduced pain, improved mobility, and increased energy for daily activities. Importantly, benefits extended beyond physical symptoms to include enhanced emotional well-being, greater self-efficacy, and strengthened social connections. These findings underscore the potential of integrating neuromodulation with mind–body interventions to address the complex, multidimensional nature of chronic pain and support meaningful improvements in daily functioning and quality of life.

## Figures and Tables

**Table 1:** Participant demographics and pain characteristics.

Participant	Biological Sex at Birth	Gender Identity	Pain Etiology	Pain Severity *	Physical Comorbidities	Psychiatric Comorbidities
A	Male	Male	Chronic low back pain, chronic neck pain	5.25	mTBI with chronic tinnitus and dizziness, sleep apnea, hyperlipidemia, Crohn’s disease	PTSD, major depressive disorder, insomnia, tobacco use disorder
B	Male	Male	Chronic low back pain, chronic genital pain	6	mTBI, essential hypertension, urinary incontinence, prostate cancer, mild diastolic left ventricular dysfunction	PTSD, major depressive disorder, personality disorder, opioid dependence on methadone, alcohol dependence in remission, tobacco use disorder
C	Male	Male	Chronic low back pain, chronic wrist pain (carpal tunnel)	6	mTBI ×2, hyperlipidemia, obesity, DMII, benign prostate hyperplasia	Insomnia
D	Male	Male	Chronic hip, knee, and shoulder pain	4	mTBI ×4, history of clavicle fracture, hip pain, knee pain	None
E	Female	Female	Chronic low back pain, chronic pelvic pain	5.5	mTBI with chronic migraines, uterine cysts status post pelvic surgeries	None
F	Male	Male	Chronic low back pain	7.25	mTBI ×3, microdiscectomy	None
G	Female	Female	Chronic low back and knee pain	4.25	mTBI ×3, h/o falls	None
H	Female	Transgender male	Chronic low back pain	6.5	mTBI ×3 with chronic migraines, GERD, fatty liver, lumbar spondylosis	PTSD, major depressive disorder with suicide attempt ×1, borderline personality disorder
I	Male	Male	Chronic low back, knee, wrist, and elbow pain	2.5 **	mTBI, obstructive sleep apnea, hyperlipidemia, pes planus, carpal tunnel	PTSD
J	Female	Female	Chronic low back, neck, and shoulder pain	6.75	mTBI ×4, obstructive sleep apnea, essential hypertension, colon polyps, hypothyroidism, carpal tunnel syndrome, discectomy	None
Average Pain Severity of All Participants *				5.4		

Abbreviations: PTSD, post-traumatic stress disorder; mTBI, mild traumatic brain injury; DMII, type 2 diabetes mellitus; GERD, gastroesophageal reflux disease. * Pain severity: Defined as the average score for questions 3 through 6 [pain at its worst (Q3), least (Q4), average (Q5) in the last week, and current (Q6)] on the Brief Pain Inventory. ** Participant verbally noted on the day of assessment that his worst typical pain was 7/10; however, at the time of written assessment, he reported he was “not in a flare up” and scored himself as noted.

**Table 2:** Quantitative pre-post change.

Measure	Change (SD)	t-Statistic	*p* Value	95% CI
TBI-QoL				
Ability	−1.66 (1.82)	−0.91	0.385	(−5.78, 2.46)
Anger	0.59 (1.90)	0.31	0.076	(−3.71, 4.89)
Anxiety	−1.29 (1.38)	−0.929	0.379	(−4.49, 1.91)
General Cognition	2.93 (2.54)	1.15	0.278	(−2.81, 8.67)
Communication	2.42 (2.39)	1.01	0.386	(−5.20, 10.04)
Depression	−2.07 (2.08)	−0.99	0.346	(−6.78, 2.64)
Emotional and Behavioral Dyscontrol	−0.82 (2.13)	−0.385	0.709	(−5.63, 3.99)
Executive Function	1.42 (2.16)	−0.66	0.528	(−3.47, 6.31)
Fatigue	−4.33 (1.55)	−2.78	0.021 *	(−7.85, −0.81)
Grief and Loss	−3.62 (1.22)	−2.95	0.016 *	(−6.39, −0.85)
Headache	0.75 (1.12)	0.667	0.522	(−1.79, 3.29)
Independence	2.3 (1.13)	2.03	0.072	(−0.26, 4.86)
Mobility	1.03 (1.49)	0.689	0.507	(−2.35, 4.41)
Pain Interference	−0.84 (2.24)	−0.38	0.716	(−5.90, 4.22)
Positive Affect and Well-Being	2.98 (1.94)	1.54	0.159	(−1.41, 7.37)
Resilience	2.71 (1.3)	2.08	0.067	(−0.23, 5.65)
Self-Esteem	1.95 (1.70)	1.14	0.282	(−1.90, 5.80)
Stigma	−0.50 (1.34)	−0.37	0.72	(−3.55, 2.55)
Mayo Portland Adaptability Index				
Ability	−5.8 (2.69)	−2.16	0.059	(−11.88, 0.28)
Adjustment	−5.8 (2.93)	−1.97	0.079	(−12.44, 0.84)
Participation	1.18 (4.01)	0.29	0.777	(−8.07, 10.43)
Total	−5.02 (4.98)	−1.00	0.343	(−16.52, 6.47)

Note: * is *p* < 0.005.

**Table 3:** Qualitative Codebook used for interviews.

Theme	Sub-Theme	Codes
Self-Management	Emotional Health	Confidence
		Motivation
		Self-efficacy
	Social Health	Community
		Validation
		Social roles
Pain Management	Physical Health	Movement
		Mobility/flexibility
		Overall well-being
		Subtle functional changes
		Increased energy
	Internal Toolkit (Cognitive Health)	Breathing/mindfulness
		External tools
		Ownership of ability
		Other pain management options

**Table 4:** Triangulation results.

Triangulation Category	Domain	Quantitative Finding	Qualitative Finding	Interpretation
Convergence	Emotional Health	TBI-QoL Fatigue (*p* = 0.021) and Grief/Loss (*p* = 0.016) significantly improved	Participants described reduced stress and improved outlook	Both methods capture emotional health improvements, confirming intervention benefits
Complementarity	Internal Toolkit	MPAI-4 Ability and Adjustment subscales showed >5-point clinically meaningful change (not statistically significant)	Participants described practical use of mindfulness, breathing, and coping strategies to regain control	Quantitative tools detect change, while qualitative data explains mechanisms of functional improvement
Divergence	Social Health	No significant change in social participation subscales	Participants highlighted peer validation, family connection, and gratitude for social support	Standardized measures under-detected meaningful improvements in social belonging and validation
	Self-Efficacy	TBI-QoL Resilience subscale trended positively but was not significant	Participants reported enhanced resilience, confidence, and empathy	Subjective resilience not fully captured by quantitative scales. This is potentially due to a lack of statistical power.

## Data Availability

Due to ethical restrictions and participant confidentiality, the data supporting this study are not available for public release. Access can be requested for qualified researchers through the VA data steward process.

## Supplementary Materials

The following supporting information can be downloaded at: https://www.mdpi.com/article/10.3390/ijerph23070872/s1, Qualitative Interview.

## Abbreviations

The following abbreviations are used in this manuscript:

mTBI + CP	Mild traumatic brain injury and chronic pain
iTBS	Intermittent theta burst stimulation
TMS	Transcranial magnetic stimulation
LYB Yoga	LoveYourBrain Yoga
TBI-QoL	Traumatic Brain Injury Quality of Life
MPAI-4	Mayo Portland Adaptability Inventory-4
rTMS	Repetitive transcranial magnetic stimulation
PTSD	Post traumatic stress disorder
