# Supplementary material for: Perspectives Among Veterans with Chronic Pain and Co-Occurring Mild Traumatic Brain Injury: Mixed-Method Findings from a Neuromodulation and Yoga Intervention
Source: Int J Environ Res Public Health. 2026 Jul 3;23(7):872. doi: 10.3390/ijerph23070872 (PMC13410723; doi:10.3390/ijerph23070872)
Supplement: Supplementary file 1 [file ijerph-23-00872-s001.zip › ijerph-4278252-supplementary.pdf]

Supplementary material

Interview Guide

Introduction: Thank you for agreeing to participate in this interview. During our interview, we would like to talk with you about your experience in the intermittent theta burst stimulation and yoga program that you participated in as part of our study. The interview should take about half an hour to complete. You can take a break or stop the interview at any time. We would like to audio-record this interview to ensure we capture what you say to the best of our ability. Is that okay with you? Do you have any other questions before we begin your interview?

First, it would be great if you could tell us a little bit about yourself.

How long have you been receiving care through VA? What kind of services have you received?

Now we'd like to talk with you about the yoga program that you participated in as part of our research study. As a reminder, your participation involved coming to the VA once a week for 6 weeks, receiving transcranial magnetic stimulation, and participating in a yoga class.

Can you please tell me why you decided to participate in our study?

Was the experience what you expected it to be? How was it similar? What about different?

Can you please tell me a little bit about what you thought of the intermittent theta burst stimulation before participating in a yoga class? What did you like about it? What did you not like?
Once you received intermittent theta burst stimulation, did anything you thought about it change? What did you like about it? What did you not like?
How could we have made the experience better?

[After reading the prompt about each of the classes (see below), the researcher will ask:]

What do you remember about that session? What did you like most (i.e., about the breathing, yoga, quote, meditation, discussion)? What would you change (i.e., about the breathing, yoga, quote, meditation, discussion)?

The first session introduced a breathing exercise where you laid on your back and used counting to regulate your breath, a series of gentle yoga poses, and a meditation where you moved your awareness throughout different parts of your body. The quote was about how yoga allows us to rediscover a sense of wholeness if our lives. The discussion asked everyone to share what being whole means to you and how you felt moving around that day.

The second session included a similar breathing exercise, gentle yoga poses, and introduced a new meditation to feel peaceful. The quote was about finding peace and balance in challenging times. The discussion was a Mindfulness Eating Exercise with a raisin.

The third session included a similar breathing exercise, gentle yoga poses, and introduced a new meditation about finding love within. The quote was about the importance of believing that things will work out. The discussion asked everyone to share one positive and one challenging aspect of their TBI and/or chronic physical pain.

The fourth session introduced a new breathing exercise where you make a sound on your exhale as if you were fogging a mirror (Ujjayi breath) and offered new gentle yoga poses. The meditation was about feeling yourself as a mountain. The quote was about resilience. The discussion described the difference between resilience and recovery and asked everyone to share one thing that has helped them become more resilient following their TBI.

The fifth session included a similar breathing exercise, gentle yoga poses, and introduced a mantra meditation. The quote was about the importance of community. The discussion asked everyone to share something interesting about themselves with the group, in the form of a game called 'Truths and Tales'.

The last session included a similar breathing exercise, gentle yoga poses, and introduced a meditation about compassion. The quote was about gratitude. The discussion asked everyone to share one takeaway from their experience in the series that they are grateful for.

Outcomes and Suggestions for Improvement

Now we would like to talk with you about how you think participating in the program impacted you, and your thoughts about how we could make the program better for Veterans who participate in the future.

What were some of the changes you noticed in yourself from participating? (e.g., Physical? Emotional? Social?). How did these changes line up with what you expected to feel after participating? Can you please tell me a little bit more about that?

> How do you think getting transcranial magnetic stimulation impacted the effects of doing yoga for you?

Overall, what did you find most helpful about participating?

Overall, what do you think could be improved about the intervention?

> How could we improve the way we delivered the TMS?
>
> How could we improve the yoga program?

Is there anything else you'd like to share?
